# Evaluation of Nafamostat as Chemoprophylaxis for SARS-CoV-2 Infection in Hamsters

**DOI:** 10.3390/v15081744

**Published:** 2023-08-15

**Authors:** Megan Neary, Joanne Sharp, Eduardo Gallardo-Toledo, Joanne Herriott, Edyta Kijak, Chloe Bramwell, Helen Cox, Lee Tatham, Helen Box, Paul Curley, Usman Arshad, Rajith K. R. Rajoli, Henry Pertinez, Anthony Valentijn, Kevin Dhaliwal, Frank Mc Caughan, James Hobson, Steve Rannard, Anja Kipar, James P. Stewart, Andrew Owen

**Affiliations:** 1 Department of Pharmacology and Therapeutics, Institute of Systems, Molecular and Integrative Biology, University of Liverpool, Liverpool L3 5TR, UKmomeejl2@liverpool.ac.uk (J.S.); e.gallardo@liverpool.ac.uk (E.G.-T.); e.kijak@liverpool.ac.uk (E.K.);; 2 Centre of Excellence in Long-Acting Therapeutics (CELT), University of Liverpool, Liverpool L3 5TR, UK; 3 Translational Healthcare Technologies Group, Queen’s Medical Research Institute, University of Edinburgh, Edinburgh EH10 5HF, UK; 4 Victor Phillip Dahdaleh Heart and Lung Research Institute, Department of Medicine, University of Cambridge, Cambridge Biomedical Campus, Papworth Road, Cambridge CB2 1BN, UK; 5 Department of Infection Biology & Microbiomes, Institute of Infection, Veterinary and Ecological Sciences, University of Liverpool, Liverpool L3 5TR, UK; anja.kipar@uzh.ch (A.K.);; 6 Laboratory for Animal Model Pathology, Institute of Veterinary Pathology, Vetsuisse Faculty, University of Zurich, 8057 Zurich, Switzerland

**Keywords:** SARS-CoV-2, nafamostat, chemoprophylaxis

## Abstract

The successful development of a chemoprophylaxis against SARS-CoV-2 could provide a tool for infection prevention that is implementable alongside vaccination programmes. Nafamostat is a serine protease inhibitor that inhibits SARS-CoV-2 entry in vitro, but it has not been characterised for chemoprophylaxis in animal models. Clinically, nafamostat is limited to intravenous delivery and has an extremely short plasma half-life. This study sought to determine whether intranasal dosing of nafamostat at 5 mg/kg twice daily was able to prevent the airborne transmission of SARS-CoV-2 from infected to uninfected Syrian Golden hamsters. SARS-CoV-2 RNA was detectable in the throat swabs of the water-treated control group 4 days after cohabitation with a SARS-CoV-2 inoculated hamster. However, throat swabs from the intranasal nafamostat-treated hamsters remained SARS-CoV-2 RNA negative for the full 4 days of cohabitation. Significantly lower SARS-CoV-2 RNA concentrations were seen in the nasal turbinates of the nafamostat-treated group compared to the control (*p* = 0.001). A plaque assay quantified a significantly lower concentration of infectious SARS-CoV-2 in the lungs of the nafamostat-treated group compared to the control (*p* = 0.035). When taken collectively with the pathological changes observed in the lungs and nasal mucosa, these data are strongly supportive of the utility of intranasally delivered nafamostat for the prevention of SARS-CoV-2 infection.

## 1. Introduction

Chemoprophylaxis is a critical tool for many infectious diseases, and in COVID-19 it may have a particular benefit for vulnerable patients that do not maximally benefit from vaccination. Certain sectors of society either cannot or will not benefit from vaccines, and concerns around their longevity on the backdrop of new and future SARS-CoV-2 variants have been raised [1]. Therefore, effective chemoprophylactic interventions represent a complimentary tool that can be deployed alongside national and international vaccination programmes [2]. For other diseases and viruses such as malaria, tuberculosis and HIV, successful prophylactic countermeasures have been developed using small-molecule inhibitors of replication [3,4,5]. The authors postulated that the topical administration of an inhibitor of SARS-CoV-2 entry via intranasal delivery to healthy individuals may have utility in preventing transmission. Transmembrane protease serine 2 (TMPRSS2) is a protease found abundantly on the surface of cells within the respiratory tract [6] and is utilised by SARS-CoV-2 for spike (S) protein priming and activation, which enables virus entry into cells [7]. TMPRSS2 activity is essential to the pathogenesis of coronaviruses [8,9], and therefore presents a putative opportunity as a drug target. The intranasal application of nafamostat has the benefit of direct delivery to SARS-CoV-2 target cells in the upper and lower respiratory tract. Furthermore, Intranasal delivery has the practical benefit of not requiring trained medical personnel or sterile conditions, meaning an intranasally delivered SARS-CoV-2 pre-exposure prophylactic could theoretically be self-administered by users as required.

Nafamostat mesylate (nafamostat) is a serine protease inhibitor used in the treatment of pancreatitis [10], and has been demonstrated to bind and inhibit TMPRSS2 and block SARS-CoV-2 entry in vitro [11]. Nafamostat is also hypothesised to have a secondary effect upon thrombotic complications in COVID-19, representing markers of severe disease that are linked to multi-organ failure and mortality [12]. Nafamostat may inhibit platelet activation, resulting in the subsequent inhibition of neutrophil extracellular traps and thus the direct activation of the intrinsic pathway. When activated alongside other pathways during SARS-CoV-2 infection, it may result in a prothrombotic state [12]. 

The current study sought to assess the potential efficacy of intranasal nafamostat in preventing the airborne transmission of SARS-CoV-2 from infected to uninfected Syrian Golden hamsters. The overarching aim was to provide preclinical data to support or refute the utility of nafamostat as a chemoprophylactic intervention for SARS-CoV-2.

## 2. Materials and Methods

### 2.1. Materials

Phosphate-buffered saline (PBS), foetal bovine serum (FBS) and 1% penicillin/streptomycin were purchased from Merck (Rahway, NJ, USA). High-glucose Dulbecco’s modified Eagle’s medium (DMEM) and Dulbecco’s PBS were purchased from Gibco^TM^C (Waltham, MA, USA). In addition, 10% neutral buffered formalin solution and 2.3% crystal violet solution were purchased from SIGMA (St. Louis, MO, USA), and 2% UltraPure LMP Agarose was purchased from Invitrogen (Waltham, MA, USA). Male Syrian Golden hamsters were purchased from Janvier Labs (Essex, UK). Further, 1 mL Amies Regular flocked swabs were purchased from Appleton Woods. Transmission cages were purchased from Techniplast UK Ltd. (Leicester, UK). The GoTaq^®^ Probe 1-Step RT-qPCR System was purchased from Promega (Fitchburg, WI, USA). The SARS-CoV-2 (2019-nCoV) CDC qPCR Probe Assay and CDC RUO 2019-nCoV_N_Positive Control were purchased from IDT (Newark, NJ, USA). TRIzol reagent, GlycoBlue^TM^, Phasemaker^TM^ tubes, Nanodrop and TURBO DNA-free^TM^ kits were purchased from Thermo Fisher (Waltham, MA, USA). A bead mill homogeniser was purchased from Fisher Scientific (Waltham, MA, USA). Precellys CKmix lysing tubes were purchased from Bertin Instruments. A Qtower^3^ Real-Time PCR Detector was purchased from Analytik Jena (Jena, Germany). For immunohistology, the rabbit anti-SARS-CoV nucleoprotein antibody was purchased from Rocklands (British CO, Canada), the peroxidase blocking buffer and the Envision + System HRP Rabbit as well as diaminobenzidine were purchased from Agilent DAKO (Carpinteria, CA, USA), and the Tissue-Tek Film for cover slipping was purchased from Sysmex (Hyogo, Japan).

### 2.2. Virus Isolates

A PANGO lineage B strain of SARS-CoV-2 (hCoV-2/human/Liverpool/REMRQ0001/2020) was used within this study. The virus was cultured from a nasopharyngeal swab collected from a patient in Liverpool in March 2020, and passaged in Vero-E6 cells. Direct RNA sequencing was previously performed and an in-house script was used to check for deletions in the mapped reads as described previously [13]. The Illumina reads were mapped to the England/2/2020 genome using HISAT and the consensus genome was called using an in-house script based on the dominant nucleotide at each location on the genome. The sequence has been submitted to GenBank, accession number MW041156.

### 2.3. Animal Study Design

All work involving SARS-CoV-2 was performed under containment level 3 (CL3) by staff equipped with respirator airstream units with a filtered air supply. Prior to the start of the study, all risk assessments and standard operating procedures were approved by the University of Liverpool Biohazards Sub-Committee and the UK Health and Safety Executive.

All animal studies were conducted in accordance with UK Home Office Animals Scientific Procedures Act (ASPA, 1986). Additionally, all studies were approved by the local University of Liverpool Animal Welfare and Ethical Review Body and performed under UK Home Office Project License PP4715265.

A random block study design was selected for the animal study. Hamsters were randomly assigned into groups of four and acclimatised for 7 days prior to study initiation. Each cage of four animals was then randomly assigned to a treatment group. The hamster selected for virus inoculation, to be designated as the donor hamster, was random in each cage. All researchers completing the viral RNA quantification, plaque assays, and histology and immunohistology analysis were blinded to the sample identifiers and treatment groups during analysis, with a separate unblinded researcher interpretating the results and completing the statistical analysis.

Male Syrian Golden hamsters (80–100 g; Janvier Labs) were housed in individually ventilated cages with environmental enrichment under SPF barrier conditions and a 12 h light/dark cycle at 21 °C ± 2 °C. Free access to food and water was provided throughout the study. All animals were weighed and monitored daily throughout the experiment.

#### Assessment of Wuhan SARS-CoV-2 Airborne Transmission in Nafamostat-Treated Syrian Golden Hamsters

**Figure 1:**
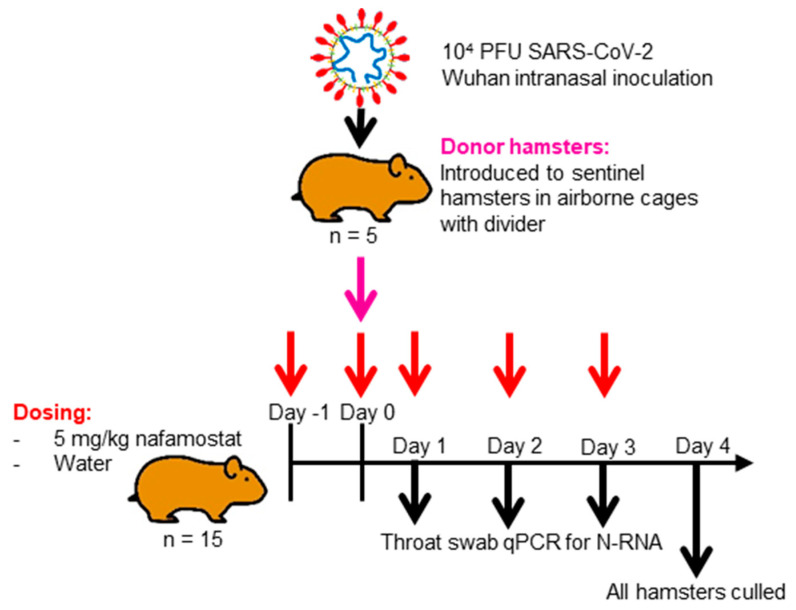
Diagrammatic representation of the experimental design employed. Study of nafamostat as chemoprophylaxis against airborne Wuhan SARS-CoV-2 infection. Sentinel hamsters were dosed intranasally twice daily with either 5 mg/kg nafamostat or water for 24 h prior to being cohoused separately in a divided cage with a hamster which was untreated and had been inoculated with Wuhan SARS-CoV-2. All animals were then throat swabbed for 4 days before study termination.

To assess the utility of nafamostat as a chemoprophylaxis against Wuhan SARS-CoV-2, as shown in Figure 1, 15 naïve hamsters in each group were intranasally dosed with 50 µL of water (control) or 5 mg/kg nafamostat in water twice in 24 h prior to being cohoused with an infected hamster, and then were intranasally dosed twice daily for four days. These animals were known as the sentinel hamsters. Following 24 h cohabitation, an untreated hamster in each group was anaesthetised under 3% isoflurane and inoculated intranasally with 100 µL of 1 × 10^4^ PFU of SARS-CoV-2 in PBS. These animals are henceforth referred to as the donor hamsters. Each study group consisted of 5 cages housing 3 treated hamsters and 1 donor hamster. The post-inoculation hamsters were housed in techniplast GR1800DIV cages with a plastic perforated divider that allowed airflow from one side to the other. In each treatment group, the donor hamster was cohoused within the same cage as the treated naïve hamsters, but was physically separated by the plastic perforated barrier to prevent contact transmission. Throat swabs were taken from all animals on days 1, 2, 3 and 4 post-inoculation of the donor hamster. The 15 naïve hamsters in each group continued their respective dosing for 4 days after donor inoculation (day 0) before the treatment was ended, and all hamsters were culled via a lethal intraperitoneal injection of pentobarbitone, which was followed by a cardiac puncture and immediate exsanguination from the heart. Samples from the right lung and nasal turbinates were then harvested and stored at −80 °C for the downstream PCR and plaque assay. Heads and left lungs were collected and fixed in 10% buffered formalin for histological analysis.

### 2.4. Quantification of Viral RNA via qPCR

A section of the dissected right lung lobe and nasal turbinate material was homogenised twice in 1 mL of TRIzol reagent (Thermo Fisher, Waltham, MA, USA) using a bead mill homogeniser (Fisher Scientific, Waltham, MA, USA) and Precellys CKmix lysing tubes (Bertin Instruments, Montigny-le-Bretonneux, France) at 3.5 m per second for 30 s. The resulting lysate was centrifuged at 12,000× *g* for 5 min at 4 °C. Throat swab media (260 µL) was added to 750 µL of the TRIzol LS reagent (Thermo Fisher). The clear supernatants were transferred to Phasemaker^TM^ tubes (Thermo Fisher) and processed as per the manufacturer’s instructions to separate total RNA from the phenol-chloroform layer. Subsequently, the recovered RNA was precipitated using GlycoBlue^TM^ according to the manufacturer’s instructions (Thermo Fisher), and was washed and solubilised in RNAse-free water. The RNA was quantified and quality assessed using a Nanodrop (Thermo Fisher). Samples were diluted to 200 ng/mL in 60 µL of RNAse-free water. The resulting RNA samples were DNAse treated using the TURBO DNA-free^TM^ kit according to the manufacturer’s instructions (Thermo Fisher). The DNAse-treated RNA was stored at −80 °C prior to downstream analysis.

The viral RNA derived from hamster lung, nasal turbinate and throat swabs was quantified using a protocol adapted from the CDC 2019-Novel Coronavirus (2019-nCoV) Real-Time PCR Diagnostic Panel [14] using the GoTaq^®^ Probe 1-Step RT-qPCR System (Promega, Fitchburg, WI, USA). For quantification of SARS-CoV-2 using the nCoV assay, the N1 primer/probe mix from the SARS-CoV-2 (2019-nCoV) CDC qPCR Probe Assay (IDT) was selected. A standard curve was prepared (200,000—2 copies/reaction) via a 10-fold serial dilution of the CDC RUO 2019-nCoV_N_Positive Control (IDT). DNAse-treated RNA at 200 ng/mL or dH_2_O was added to the appropriate wells, producing final reaction volumes of 20 µL. The prepared plates were run using a Qtower^3^ Real-Time PCR Detector (Analytik Jena). The thermal cycling conditions for the qRT-PCR reactions were: 1 cycle of 45 °C for 15 min and 1 cycle of 95 °C for 2 min, followed by 40 cycles of 95 °C for 3 s and 55 °C for 30 s.

The quantification of 18S RNA utilised previously described primers and probe sequences [15], which were used at 300 nM and 200 nM, respectively (IDT), using the GoTaq^®^ Probe 1-Step RT-qPCR System (Promega, Fitchburg, WI, USA). Methods for the generation of the 18S standards have been outlined previously [16]. The PCR product was serially diluted to produce a standard curve in the range of 5 × 10^8^, or 5 copies/reaction, via a 10-fold serial dilution. DNAse-treated RNA at 200 ng/mL or dH_2_O was added to the appropriate wells, producing final reaction volumes of 20 µL. The prepared plates were run using a qTOWER^3^ Real-Time PCR Detector (Analytik Jena, Jena, Germany). The thermal cycling conditions for the qRT-PCR reactions were: 1 cycle of 45 °C for 15 min and 1 cycle of 95 °C for 2 min, followed by 40 cycles of 95 °C for 3 s and 55 °C for 30 s. N-RNA data were normalised to 18S data for subsequent quantitation. The limit of detection (LOD) for the assay was defined as an N-RNA value of ≤2 copies/reaction and a PCR Ct value cut off of ≥32 cycles. These were selected based on previously published data which demonstrated that PCR Ct values between 17 and 32 represent culturable virus amounts, and are assumed to be infectious [17,18].

### 2.5. Plaque Assay

A section of the dissected right lung lobe was homogenised in Precellys^®^ tubes in 1 mL of Dulbecco’s PBS (GibcoTM, Waltham, MA, USA). Samples were homogenised twice using a bead mill homogeniser (Fisher Scientific) at 3.5 m per second (m/s) for 30 s. Samples were then stored at −80 °C for downstream analysis in plaque assays.

Vero-E6 cells (passages 11 to 16) were maintained in high-glucose DMEM (Gibco^TM^) that was supplemented with 10% FBS and 1% penicillin/streptomycin at 37 °C and 5% CO_2_. Vero-E6 cells at an 80–90% confluency where seeded at 3 × 10^5^ cells/well in 24-well plates and incubated overnight at 37 °C and 5% CO_2_. Once 100% confluency of the cells was confirmed in each well, the plates were transferred to CL3, and the plaque assay was completed. 

A serial dilution of homogenised tissue samples was prepared in maintenance media (high-glucose DMEM, 2% FBS) in the range of 1–10^−7^ of the virus titre within the sample.

Then, 100 µL of the serially diluted tissue samples were added to each well of the Vero-E6 cell 24-well plate in duplicate and incubated for one hour at 37 °C and 5% CO_2_. Next, 500 µL of freshly prepared overlay (maintenance media and 2% UltraPure LMP Agarose (Invitrogen), 4:1) was added to each well and cells were incubated for 72 h at 37 °C and 5% CO_2_. After the incubation period, cells were fixed with 10% neutral buffered formalin solution (SIGMA) for 30 min and stained with 2.3% crystal violet solution (SIGMA). 

In order to determine the plaque-forming units per mL (PFU/mL), the number of formed plaques per well were counted manually and the following formula applied:

(# Plaques)/(d × V) = PFU/mL

where d = the dilution factor, and V = the volume of diluted virus added to the well (mL).

### 2.6. Histological and Immunohistological Analyses

From all animals the left lung and head were fixed in 10% buffered formalin for 48 h and then stored in 70% ethanol until further processing. Two longitudinal sections were prepared from the lung and were routinely embedded in paraffin wax. Cross sections of the nose, taken using a diamond saw (Exakt 300; Exakt), were prepared in approximately 1.5 mm thick slices (tip of the nose to the level of the olfactory bulb). Sections were gently decalcified in RDF (Biosystems, Foster City, CA, USA) for 10 days at room temperature (RT) and on a shaker, and then were embedded in paraffin wax. Consecutive sections (3–5 µm) were prepared and stained with haematoxylin eosin (HE) for histological examination or subjected to immunohistological staining to detect the SARS-CoV-2 antigen (performed in an autostainer; Agilent DAKO, Carpinteria, CA, USA), using the horseradish peroxidase (HRP) method and rabbit anti-SARS-CoV nucleocapsid protein (Rockland) as previously described [19]. Briefly, sections were deparaffinised and rehydrated with graded alcohol. Antigen retrieval was achieved via 20 min incubation in citrate buffer (pH 6.0) at 98 °C in a pressure cooker. This was followed by incubation with the primary antibody (diluted 1:3000 in dilution buffer; Agilent Dako) overnight at 4 °C, a 10 min incubation in RT with peroxidase blocking buffer (Agilent Dako) and a 30 min incubation in RT with the Envision + System HRP Rabbit (Agilent Dako, CA, USA). The reaction was visualised with diaminobenzidine (DAB; Dako) for 10 min in RT. After counterstaining with haematoxylin for 2 s, sections were dehydrated and placed on a coverslip with Tissue-Tek Film (Sysmex).

### 2.7. Statistical Analysis

Prior to the study, a power calculation was conducted for a two-sided unpaired *t*-test to determine the number of experimental units per study group required to complete a comparison of the lung viral RNA values, which quantified for the water-treated control group and the nafamostat-treated group a power of 0.8 and a significance level of 0.05. A minimum effect size was determined as a 2-fold difference in lung viral RNA, with a standard deviation of 0.38 derived from previous comparable studies conducted in house. An n number of 4 per group was calculated. The power calculation was completed using the NC3Rs Experimental Design Assistant. All sentinel hamsters where housed 3 per cage with a single donor hamster, with a total of five cages per group (15 sentinels and 5 donor hamsters per group total). 

A 2-way mixed-effect ANOVA with a Bonferroni correction to correct for multiple comparisons was applied to compare the percentage weight change or viral RNA quantified in the throat swabs by qPCR between the water-treated control and the nafamostat-treated group over the time course of the study. An unpaired t-test was used to compare the differences in the viral RNA load between the water-treated control group and the nafamostat-treated group’s throat swabs, and their lung and nasal turbinate samples. A *p*-value of ≤0.05 was taken as statistically significant. All statistical analyses were completed using GraphPad Prism version 8.3.0.

## 3. Results

### 3.1. Changes in Body Weight

**Figure 2:**
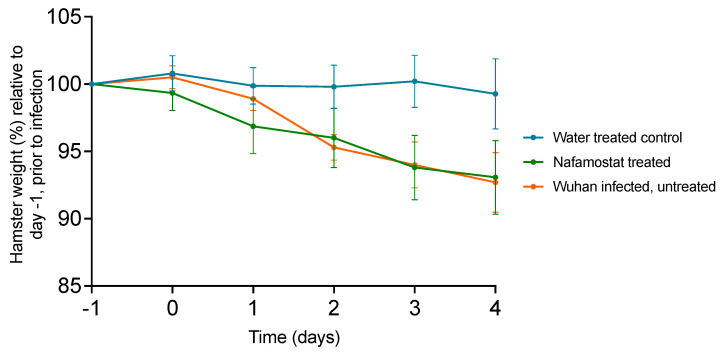
Hamster weights separated by group and inoculation status. All hamsters within each treatment group (n = 15), as well as the donor hamster (inoculated with Wuhan SARS-CoV-2) cohoused with each group (n = 10), were weighed at 24 h intervals up to the study endpoint on day 4. All weights are shown as a percentage of the initial weight recorded at baseline on day −1 of the study. Error bars represent the standard deviation for each study group at each timepoint.

Body weight changes in each group over the course of the experiment are shown in Figure 2. In the donor hamsters, a loss of body weight was observed, starting from day 1 post-infection and reaching an average of 7.3% by day 4. In the sentinel hamsters, weight loss was only observed in the nafamostat-treated group, where it started at day 1 and was progressive but did not exceed 6%. In comparison to the weight at baseline on day −1 over the full study time course, the weight loss was significant (*p* ≤ 0.0001) in both the donor animals and the nafamostat-treated group at day 4 compared to the water-treated control group (1% at day 4). 

### 3.2. Viral RNA Quantification from Swab and Tissue Samples 

**Figure 3:**
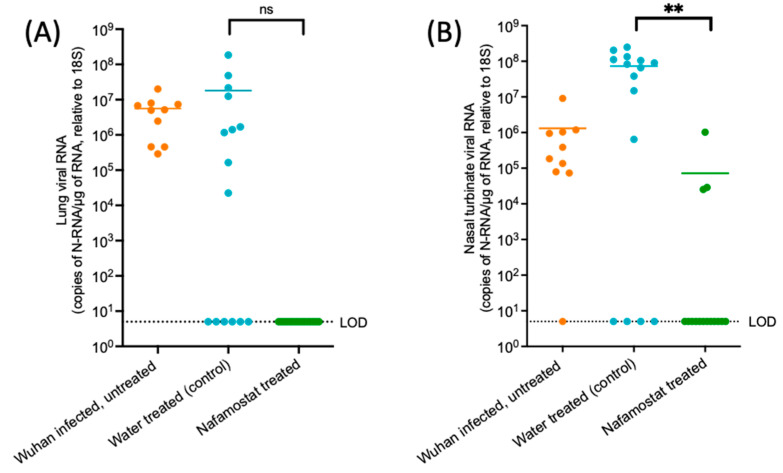
Viral quantification of SARS-CoV-2 N-RNA within lung and nasal turbinate samples obtained from the study groups at day 4. Quantification of SARS-CoV-2 N-RNA in (**A**) lung and (**B**) nasal turbinate samples from the Wuhan infected, untreated (donor) hamsters (n = 10), the water-treated hamsters (n = 15) and the nafamostat-treated hamsters (n = 15) at day 4. An unpaired *t*-test was used to determine statistical significance (*p* ≤ 0.05) between the water-treated control group and the nafamostat-treated group. ns = not statistically significant. ** = statistically significant (*p* ≤0.01 and > 0.001).

**Figure 4:**
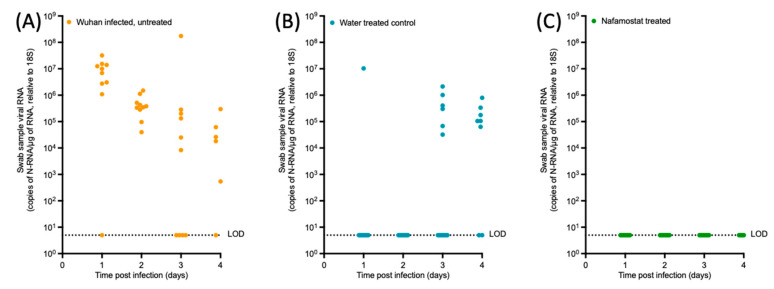
SARS-CoV-2 RNA quantified from throat swab samples taken from each study group. SARS-CoV-2 viral N-RNA quantified from throat swab samples taken at day 1, 2, 3 and 4 of the study from the (**A**) Wuhan infected, untreated (donor) control hamsters (n = 10), (**B**) water-treated hamsters (n = 15) or (**C**) nafamostat-treated hamsters (n = 15). LOD = limit of detection. A two-way ANOVA was applied to determine statistical significance between the water-treated control group (**B**) and the nafamostat-treated group (**C**) at each timepoint; no significant difference was observed (*p* = 0.5163).

The quantification of virus N-RNA from the lung and nasal turbinate samples as well as throat swabs is shown in Figure 3 and Figure 4, respectively. At 4 dpi, viral N-RNA was readily detected in the lungs of all intranasally infected donor animals. It was also detected in 60% of the water-treated sentinel animals and at similar levels, but it was not detected in any of the lungs of the nafamostat-treated animals. Of note, three of the six sentinel hamsters with undetectable viral N-RNA in the lungs in the water-treated group were housed in the same cage. Overall, there was no significant difference between the viral N-RNA quantified in the lungs of the water-treated control group and the nafamostat-treated group (*p* = 0.154).

The viral N-RNA levels in the nasal turbinate samples were overall higher in the water-treated animals than in the donor animals, but the difference was not significant. However, they were significantly lower in the nafamostat group compared to the water-treated controls (*p* = 0.0012), and in 4 and 12 animals, respectively, the levels were below the LOD for the PCR assay.

In the donor animals, viral N-RNA was detected in the throat swab samples throughout the entire sampling period: in 9 of the 10 hamsters on day 1, in all animals on day 2, in 6 on day 3, and in 8 on day 4. In the water-treated control group, viral N-RNA was detectable in the throat swabs of 1 sentinel animal on day 1, 6 sentinels on day 3 and 12 sentinels on day 4. In contrast, it was below the LOD in the nafamostat-treated group for the entirety of the study. However, no statistically significant difference was observed between the water-treated control group and the nafamostat-treated animals over the study time course (*p* = 0.5163). Peak viral N-RNA relative to 18S was observed at day 3 in the donor group and the water-treated control group, with an average value of 17,479,581.52 copies of N-RNA/µg of RNA relative to 18S and 263,777.98 copies of N-RNA/µg of RNA relative to 18S, respectively.

### 3.3. Live Virus Quantification Obtained through Plaque Assay

**Figure 5:**
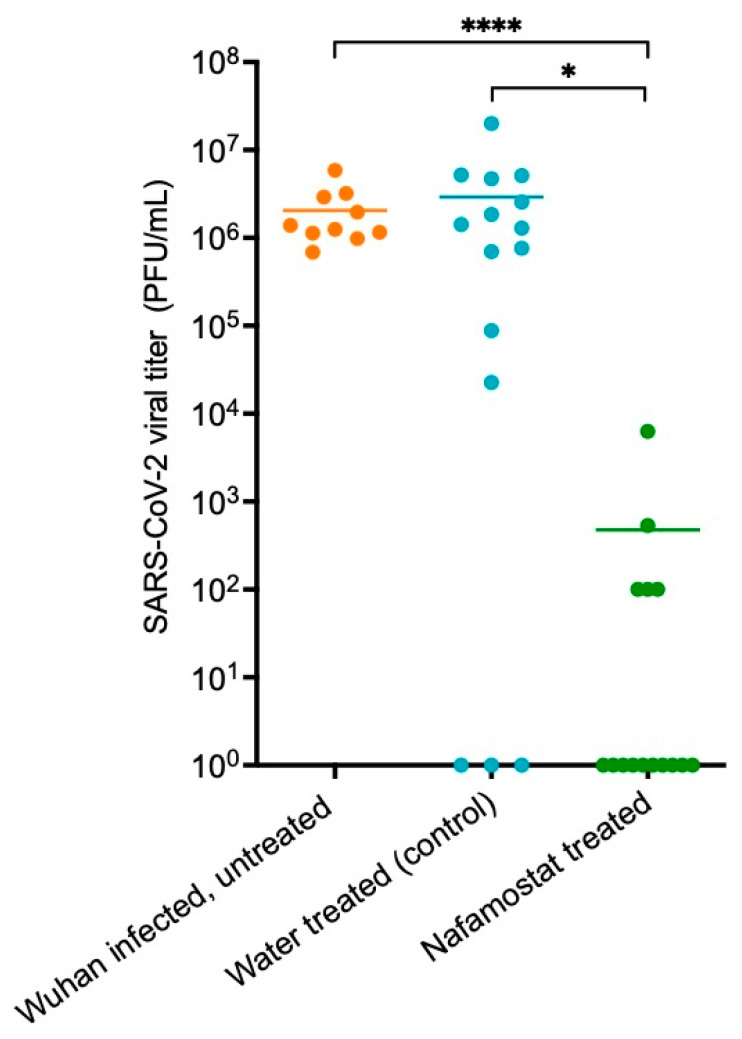
SARS-CoV-2 titres quantified from tissue samples from the donor, nafamostat- or water-treated hamsters. An unpaired *t*-test was used to determine statistical significance (*p* ≤ 0.05) between the water-treated control group and the nafamostat-treated group. * = statistically significant (*p* ≤ 0.05 and >0.001). **** = statistically significant (*p* ≥ 0.0001).

The quantification of live SARS-CoV-2 in lung samples obtained from the donor and sentinel hamsters in the water-treated control group and the nafamostat-treated group is shown in Figure 5. The SARS-CoV-2 titres in water-treated sentinel hamsters were not significantly different from those in the donor hamsters (*p* = 0.613). They were significantly lower in the lung samples of the nafamostat-treated group compared to the water-treated controls (*p* = 0.0350) and the donor hamsters (*p* ≥ 0.0001). Furthermore, live virus was detectable in all 10 donor hamster lung samples, in 12 out of 15 samples taken from the water-treated control group, and in 5 out of 15 lung samples obtained from the nafamostat group.

### 3.4. Pathological Changes in the Lungs and Nasal Mucosa 

**Figure 6:**
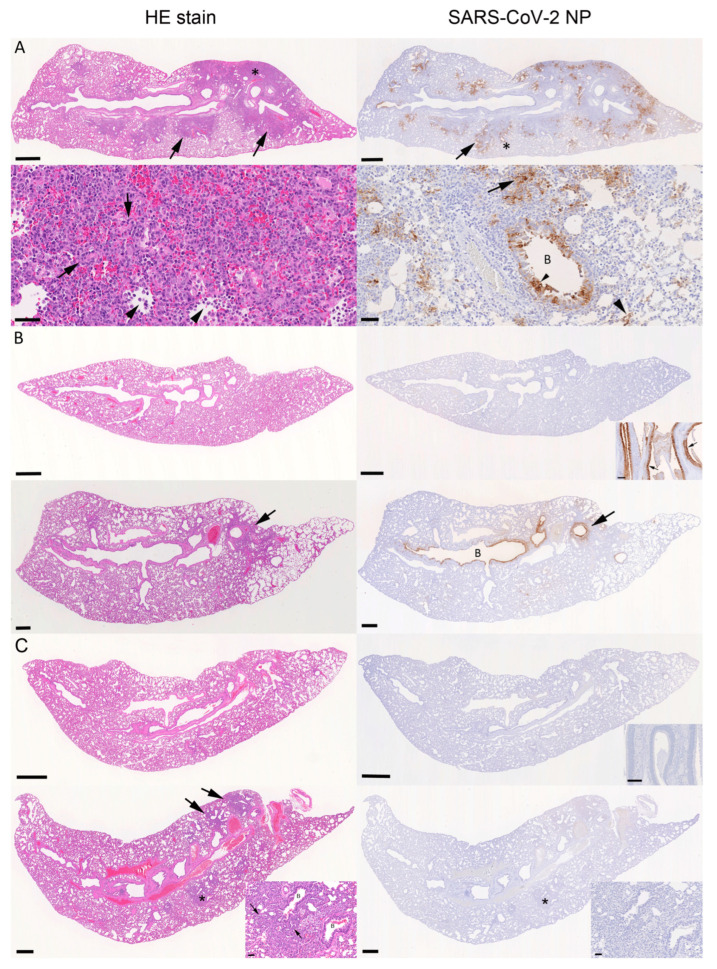
Histological findings and viral antigen expression in lungs and nose of donor; water- or nafamostat-treated hamsters examined at 4 days after intranasal infection of the donor by 10^4^ PFU of SARS-CoV-2 Wuhan. (**A**) Donor hamster (animal #6), left lung, longitudinal section. The overview of the HE-stained section shows multifocal, bronchiole-centred, consolidated areas (**top left**: arrows); a higher magnification of the focal area (*) reveals a dense leukocyte infiltration, comprising macrophages, fewer lymphocytes and a variable numbers of neutrophils, with focal desquamation of infected cells (**bottom left**: arrowheads) and mild hyperplasia of type II pneumocytes/bronchiolar epithelial cells (**bottom left**: arrows). Immunohistology shows abundant SARS-CoV-2 N expression in patches of alveoli, often in the periphery of consolidated areas (**right**: arrows). A higher magnification (*) also reveals staining in small patches of unaltered alveoli (bottom right: large arrowhead) and in bronchial epithelial cells (**bottom right**: small arrowhead). B: Bronchiole. (**B**) Water-treated sentinel animals. Top: Animal #2 (cage 1; N-RNA and protein negative in lung and nose, plaque assay negative). The lung is histologically unaltered (HE stain, **left**) and there is no obvious viral antigen expression (**right**). Inset: Nasal mucosa with focally abundant SARS-CoV-2 N positive epithelial cells. Bottom: Animal #7 (cage 3). The lung exhibits a focal area of peribronchiolar consolidation (**left**: arrow) with moderate viral antigen expression in bronchus and several bronchioles (**right**: arrow) as well as in some patches of alveoli. B: Bronchiole. (**C**) Nafamostat-treated sentinel animals. Top: Animal #6 (cage 7; N-RNA and protein negative in lung and nose, plaque assay negative). The lung is histologically unaltered (HE stain, **left**), and both lungs (**right**) and nasal mucosa (inset) do not exhibit viral antigen expression. Bottom: Animal #8 (cage 8; N-RNA and protein negative in lung and nose, plaque assay negative). The lung exhibits a few small, consolidated areas (HE stain, **left**; arrows); a higher magnification of the focal area (*) reveals hyperplasia of type II pneumocytes/bronchiolar epithelial cells (insets: arrows) with mild leukocyte infiltration. There is no evidence of viral antigen expression (**right**). B: Bronchiole. (**A**). Bars = 1 mm (**top** images) and 50 µm (**bottom** images). (**B**). Bars = 1 mm (overview images) and 50 µm (inset, nasal mucosa). (**C**). Bars = 500 µm (overview images) and 50 µm (insets).

The histological examination of the lungs in combination with the immunohistological staining for SARS-CoV-2 NP confirmed widespread lung infection in all donor hamsters (Figure 6A). Both respiratory epithelial cells in bronchi/-oles and alveolar epithelial cells (type I and II pneumocytes) were confirmed to be infected, with evidence of degeneration and desquamation of infected cells and their release into the exudate (sloughed-off infected cells in the lumen of airways; 8/10 animals), which is a source of viral shedding. Infection was associated with the activation of type II pneumocytes; variable bronchial/bronchiolar, peribronchial/peribronchiolar and parenchymal leukocyte infiltration (generally comprised of macrophages, fewer lymphocytes and variable numbers of neutrophils); and a variable degree of vasculitis. The findings are consistent with those reported in hamsters 4 days after wildtype SARS-CoV-2 infection [20]. In three animals there were changes consistent with early regenerative attempts, i.e., mild hyperplasia of type II pneumocytes/bronchiolar epithelial cells (Figure 6A); these have previously been reported to be present already at this timepoint [20,21]. All donors exhibited degeneration of respiratory epithelial cells and a variably intense neutrophil-dominated rhinitis, in which the detection of viral N-RNA and/or NP confirmed the infection. Individual animal data are provided in Supplementary Table S1.

In the water-treated sentinel hamsters, the number of infected animals, the extent of virus spread and the associated pathological changes varied between cages. In cage 1, infection of the sentinels (water-treated hamsters #1–3) did not go beyond a nasal infection in two hamsters, as shown by PCR or immunohistology, and was in combination with the absence of histological changes in the lungs (Figure 6B). This was also the case for one animal in cage 3 (water-treated hamster #8). In cage 2, the infection had reached the lungs in all sentinels (water-treated hamsters #4–6), but was generally very limited and associated with no or only minimal damage. The same was observed in two of the three sentinel animals in cages 3 (water-treated hamsters #7 and 9) and 4 (water-treated hamsters #11 and 12), and all sentinel hamsters in cage 5 (water-treated hamsters #13–15) (Figure 6B). In the third sentinel in cage 4 (water-treated hamster #10) changes very similar to those seen in the donor animals and substantial viral antigen expression were observed; the presence of some type II pneumocyte/bronchiolar epithelial cell hyperplasia even suggested early regenerative attempts. Interestingly, this animal tested negative for N-RNA but positive for live virus through the plaque assay in the contralateral lung. Individual animal data are provided in Supplementary Table S1.

Among the nafamostat-treated sentinel hamsters were two (cage 7, nafamostat-treated hamster #6, and cage 9, nafamostat-treated hamster #10) that did not exhibit any evidence of infection (nose and lung negative for both viral N-RNA and protein, and negative in the plaque assay) (Figure 6C). The remaining two sentinels in cage 9 (nafamostat-treated hamsters #11 and 12) did not exhibit histological changes, viral N-RNA or NP expression in the lungs and tested negative for live virus through the plaque assay, but were found to carry the virus in the nasal cavity, as shown by the detection of viral N-RNA. All other sentinel animals exhibited histological changes in the lung suggestive of prior infection [20,21], which is mainly represented by focal areas with leukocyte infiltrates, activated type II pneumocytes and minimal hyperplasia of type II pneumocytes/bronchiolar epithelial cells (nafamostat-treated hamsters #1–5, 7–9, and 13–15) (Figure 6C). Live virus was detected in the plaque assay of nafamostat-treated hamsters #1–5 and 7, but was undetectable in nafamostat-treated hamsters #8–15. Neither viral N-RNA nor NP were detected in the lungs or the nose of these animals apart from #1 (cage 6) and #8 (cage 8), in which each harboured rare intralesional macrophages with weak viral antigen expression in the lung.

## 4. Discussion

This study sought to determine the suitability of intranasally delivered nafamostat for use as a chemoprophylaxis against SARS-CoV-2 in an airborne transmission model in Syrian Golden hamsters, where donor and sentinel hamsters received a separate food and water supply and were physically separated by a plastic perforated barrier. The intranasal infection of Syrian Golden hamsters with SARS-CoV-2 has previously been demonstrated to result in viral titres and pathological changes similar to those seen in human patients [22]. Importantly for the current study, the TMPRSS2-mediated priming of the SARS-CoV-2 S protein was found to be largely similar in hamsters and humans [23], highlighting the similarity between the host response in hamsters and humans and supporting the use of this model for TMPRSS2-targeted therapeutics. However, it should be noted that as a rodent, the hamster is an obligate nasal breather [24] and has abbreviated bronchioles, which may result in the faster therapeutic clearance in the alveolar of hamsters compared to humans [25]. The importance of this for the translatability of the results in humans is uncertain, but may suggest a potential limitation for the use of this species to characterise intranasally delivered chemoprophylactic interventions. It is not anticipated that obligate nasal breathing would lower the efficacy of intranasally administered nafamostat; however, it may increase virus acquisition in the lungs of hamsters.

The intranasal dosing of test therapeutics enables direct drug delivery to a primary site of respiratory virus infection, which may be particularly useful for chemoprophylaxis. However, it has so far been unclear whether nafamostat delivered via this route would be sufficient to meaningfully alter the course of SARS-CoV-2 if given in a therapy model. A single-dose pretreatment of Ad5-*hACE2*-transduced mice and K18-*hACE2* mice with 3 mg/kg of nafamostat intranasally delivered prior to inoculation with SARS-CoV-2 significantly reduced the viral titres over the study time course [26]. Furthermore, a study of 420 µg/mL of nafamostat suspended within a novel lipid formulation and administered intranasally to hamsters, which was immediately followed by inoculation with SARS-CoV-2, showed a transient but significant reduction in the viral load within the nasal cavity compared to controls [27]. Unfortunately, a histopathological assessment of the animals was not undertaken. Also, it is not possible to ascertain whether the limited benefit was a result of the formulation itself, since unformulated nafamostat was not used as a control [27]. However, studies with a primary lung epithelium cell model demonstrated the low cytotoxicity of the lipid formulation up to 6 µg/mL of nafamostat, with evidence of SARS-CoV-2 inhibition at this dose [28]. Given that the systemic half-life of nafamostat after intravenous delivery is only 8 min [29], the protection reported in the present study, with twice daily administration, bodes extremely well for nafamostat reaching its target when intranasally administered. Nafamostat is not currently approved for oral or inhalational administration in human patients, so the development of a specific formulation may be needed if this approach is to be tested in humans.

Lung and nasal turbinate samples were collected at study termination in order to determine the presence or absence of the virus, as well as to analyse the pathological changes in hamsters when given nafamostat as a chemoprophylaxis compared to the donor hamsters or water-treated control group. Widespread SARS-CoV-2 infection in all donor hamsters was confirmed through the quantification of viral RNA, semi-quantitative live virus calculation via plaque assay, and immunohistological staining for the viral antigen. Similarly, all water-treated sentinel animals in four cages were found to be infected, with both nose and lungs harbouring the virus, confirming successful airborne transmission. Interestingly, transmission was less successful in cage #1, where only one sentinel hamster carried the virus in the nose and lungs; in one, only a nasal infection was confirmed, and the third tested negative throughout. It is possible that the donor animal in this cage only shed the virus intermittently or at lower levels [30]. Also, overall, viral RNA levels and the extent of viral antigen expression were lower than in the donor animals. As shown in Figure 3 and Figure 4, the lung, nasal turbinate and swab sample viral RNA at days 3 and 4 in the donor hamsters was comparable to that in the water-treated control group. These high viral loads at the study endpoint indicate a recent infection, which could be the result of airborne transmission between the sentinel hamsters, but could also have been transmitted from the sentinel hamsters back to the donor hamster. 

With the nafamostat treatment, two thirds of the sentinel animals tested negative by all approaches at 4 dpi, and there was generally no viral antigen detected. However, several of these animals showed focal type II pneumocyte/bronchiolar epithelial cell hyperplasia, which is consistent with regenerative attempts after infection, as it can be seen as early as 3 days post infection in the hamster model of COVID-19 [20,21].

Some discrepancies were observed between the results of the different approaches to detect and quantify infection. In the case of a very limited and focal rather than diffuse infection, this may in part be attributed to the sampling protocol where the left lung was processed for histology and immunohistology, and the right lung was subjected to the quantitative PCR and plaque assay. A further limitation of the present study is that intranasally nafamostat-dosed animals not cohoused with a donor hamster were not included in the study, meaning that determining if intranasal dosing alone induces histological changes in the nasal mucosa and lung is beyond the scope of this study. In three hamsters in the water-treated control group and five hamsters in the nafamostat-treated group, a N-gene PCR value below the LOD for the assay was observed alongside a positive plaque assay result. Variation in virus detection between the different analyses may be caused by the LOD cut off applied to the viral N-gene RNA quantification via PCR. For the PCR assay, an LOD was defined as an N-RNA value of ≤2 copies/reaction and the PCR Ct value cut off was 32 cycles. This LOD was determined given that PCR Ct values between 17 and 32 have been demonstrated previously to represent culturable virus amounts, which are assumed to be infectious [17,18]. The inclusion of this Ct cut off as part of the LOD may exclude quantifiable values of viral N-RNA lower than 2 copies/reaction. Consideration must also be given to the plaque assay being semi-quantitative and to its ability to exclusively highlight the presence of live virus within the lung samples. Despite these limitations, collectively these methods enable a comprehensive evaluation of the chemoprophylactic effect of intranasally administered nafamostat, which must be holistically assessed.

Of note, when using the plaque assay data, the transmission rate of SARS-CoV-2 from the donor hamsters to the water-treated control hamsters and to the nafamostat-treated hamsters was found to be 80% and 33%, respectively. A previous study which used surgical mask material to separate a donor hamster from untreated hamsters recorded a reduction in transmissibility from 60% in the no surgical mask partition group to 12.5% in the surgical mask partitioned group, which was culled at day 4 after cohousing [31]. This comparable reduction in transmissibility between the nafamostat treatment and mask wearing is of interest. In a real-world situation, mask wearing could be combined with the administration of an intranasal chemoprophylaxis, but additional studies would be needed to ascertain the benefits of such an approach. Mask use and chemoprophylaxis both have a role to play in SARS-CoV-2 disease prevention in people who are at risk of complications from disease acquisition. The development of a self-administered aerosol of nafamostat may afford additional discretion for the user and would enable protection for people with a low tolerance for wearing masks over extended periods of time.

The primary mechanism of action of nafamostat is thought to be the inhibition of the TMPRSS2 serine protease, which plays an important role in cell entry. This is important because some data have suggested differences in the importance of TMPRSS2 for the entry of different SARS-CoV-2 lineages and sub-lineages [32,33,34]. Accordingly, future clinical trials and deployment may need to consider the relative importance of TMPRSS2 for variants predominantly circulating at the time of intervention. Further studies would be beneficial to assess the pharmacokinetics of intranasally delivered nafamostat in respiratory secretions and whether the efficacy of nafamostat can be boosted through delivery with an aerosol. Overall, clear differences in the response to cohousing with the donor hamsters was observed between the water-treated control group and the nafamostat-treated hamsters (see Supplementary Table S1) in terms of viral RNA quantification, live virus calculation, and the histological and immunohistological findings. Taken collectively, these data demonstrate that 5 mg/kg of twice daily intranasally administered nafamostat can exert a chemoprophylactic effect in healthy hamsters.

## Data Availability

The data presented in this study are available upon request from the corresponding author or can be found in Supplementary Table S1.

## Supplementary Materials

The following supporting information can be downloaded at: https://www.mdpi.com/article/10.3390/v15081744/s1, Table S1: Relevant histological changes and SARS-CoV-2 nucleoprotein expression in Syrian Golden hamsters after intranasal infection with 10^4^ PFU SARS-CoV-2 Wuhan WT and euthanised at 4 days post-infection (infected hamsters #1–#10), and in hamsters that were exposed to airborne transmission (AT) and treated intranasally with water (water-treated hamsters #1 to #15) or nafamostat (nafamostat-treated hamsters #1 to #15) for 5 days. NB: The olfactory bulb was included in most sections from the head. It was consistently negative for viral antigen. AEC—alveolar epithelial cells; AM—alveolar macrophages; AT—airborne transmission; BEC—bronchiolar epithelial cells; deg—degenerate; d—day; EC—epithelial cells; HE—histological features assessed in a haematoxylin-eosin-stained section; in—intranasal; LC—lymphocyte; mf—multifocal; mode—moderate; neg—negative; NHA—no histological abnormality; NL—neutrophils; NT—nasal turbinates; LC—lymphocytes; pb—peribronchiolar; pc—pneumocytes; pos—positive; pv—perivascular; vAg—viral antigen. ^1^ PCR: copies of viral SgE-RNA/µg of RNA relative to 18S; plaque assay: SARS-CoV-2 viral titre (PFU/mL). ^2^ Leukocyte infiltration/leukocyte infiltrates: if not further specified, this implies macrophages, fewer lymphocytes and variable numbers of neutrophils.
