# Supplementary material for: Evaluation of Nafamostat as Chemoprophylaxis for SARS-CoV-2 Infection in Hamsters
Source: Viruses. 2023 Aug 15;15(8):1744. doi: 10.3390/v15081744 (PMC10458615; doi:10.3390/v15081744)
Supplement: Supplementary file 1 [file viruses-15-01744-s001.zip › viruses-2517862-supplementary.pdf]

Supplementary Table S1. Relevant histological changes and SARS-CoV-2 nucleoprotein expression in Syrian hamsters after intranasal infection with 10^4 PFU SARS-CoV-2 Wuhan WT and euthanised at 4 days post infection ("Infected #1-#10), and in hamsters that were exposed to airborne transmission (AT) and treated intranasally with water ("Water treated #1 to #15) or nafamostat (Nafamostat traeated #1 to #15) for 5 days**.**

NB: The olfactory bulb was included in most sections from the head. It was consistently negative for viral antigen.

**Animal No, treatment**	**Histological changes and viral antigen expression (lung, nasal mucosa)**	**Virology (PCR; plaque assay)**[1]
**Infected #1** (Cage 1)	**Lung**: mild to mod mf pb leukocyte infiltration[2] with mild to mod vasculitis of adjacent vessel; mod pb and mild bronchial LC-dominated leukocyte infiltration and scattered deg BEC, some leukocytes incl vacuolated macrophages and deg cells in lumen; a few areas with desquamation of AEC and AM and leukocyte infiltration<br>**vAg**: mf, partly coalescing patches of alveoli with pos AEC, pos deg cells and macrophages, particularly abundant pos cells in area with desquamative component; bronchus and several bronchioles with individual or large patches of pos BEC and some pos cells in lumen	Lung: 5010292; positive
	**Nasal mucosa (HE)**: mf deg and loss of EC, mod NL-dominated rhinitis<br>**vAg**: disseminated large patches and some individual pos EC, abundant deg pos cells in lumen	NT: 385263
Water treated #1 (Cage 1)	**Lung**: NHA<br>**vAg**: neg	Lung: <LOD; negative
	**Nasal mucosa (HE)**: NHA<br>**vAg**: neg	NT: 645444
Water treated #2 (Cage 1)	**Lung**: NHA<br>**vAg**: a few pos intact AEC	Lung: <LOD; negative
	**Nasal mucosa (HE)**: focal loss of EC and NL-dominated rhinitis<br>**vAg**: mf large patches of pos EC, disseminated some individual pos EC	NT: <LOD
Water treated #3 (Cage 1)	**Lung**: NHA<br>**vAg**: neg	Lung: <LOD; negative
	**Nasal mucosa (HE)**: NHA<br>**vAg**: neg	NT: <LOD
**Infected #2** (Cage 2)	**Lung**: bronchus with mod to marked pb and mild bronchial leukocyte infiltration and a few deg EC; abundant deg cells in lumen, adjacent one focal area with desquamation of AEC and AM and leukocyte infiltration, with activated type II pc and mild to mod vasculitis; a few bronchioles with a few deg cells and mild pb leukocyte infiltration<br>**vAg**: a few large patches of alveoli with pos AEC; bronchus and several bronchioles with patches to all pos BEC and abundant pos cells in lumen	Lung: 6758476; positive
	**Nasal mucosa (HE)**: mf deg and loss of EC, mod NL-dominated rhinitis<br>**vAg**: mf large patches of pos EC, disseminated some individual pos EC, abundant deg pos cells in lumen	NT: 9109105

Water treated #4 (Cage 2)	**Lung**: NHA<br>**vAg**: bronchus and focal area with a few bronchioles with almost all EC pos, one large adjacent patch of alveoli with pos AEC	Lung: 183931979; positive
	**Nasal mucosa (HE)**: NHA<br>**vAg**: neg	NT: 111604427
Water treated #5 (Cage 2)	**Lung**: NHA, apart from one small focal pb area with a few desquamed vacuolated macrophages and mild leukocyte infiltration<br>**vAg**: neg, apart from one bronchiole with a patch and several individual pos intact EC	Lung: 164224; positive
	**Nasal mucosa (HE)**: mild NL-dominated rhinitis<br>**vAg**: mf large patches of pos EC, some individual pos EC in apical mucosa	NT: 247899202
Water treated #6 (Cage 2)	**Lung**: NHA, apart from bronchus with a few deg cells and mild pb leukocyte infiltration<br>**vAg**: bronchus and several bronchioles with large patches to all pos EC; a few small random patches of alveoli with pos AEC	Lung: 12468206; positive
	**Nasal mucosa (HE)**: mild focal NL-dominated rhinitis<br>**vAg**: mf large patches of pos EC, disseminated some individual pos EC	NT: 66117868
**Infected #3** (Cage 3)	**Lung**: mild to mod mf pb leukocyte infiltration with mild to mod vasculitis of adjacent vessel; mod pb and mild bronchial LC-dominated leukocyte infiltration and scattered deg BEC, abundant leukocytes incl vacuolated macrophages and deg cells in lumen; a few areas with desquamation of AEC and AM and leukocyte infiltration<br>**vAg**: mf, partly coalescing patches of alveoli with pos AEC, also with pos deg cells and macrophages; bronchus and bronchioles with individual or patches of pos BEC and abundant pos cells in lumen	Lung: 2453408; positive
	**Nasal mucosa (HE)**: mf deg and loss of EC, severe NL-dominated rhinitis<br>**vAg**: mf large patches of pos EC, disseminated some individual pos EC, abundant deg pos cells in lumen	NT: <LOD
Water treated #7 (Cage 3)	**Lung**: NHA, apart from one bronchiole with deg EC and adjacent mild focal vasculitis<br>**vAg**: many bronchioles with large patches to all pos EC; small patches of adjacent alveoli with pos AEC	Lung: 1686340; positive
	**Nasal mucosa (HE)**: severe NL-dominated rhinitis<br>**vAg**: mf large patches of pos EC, disseminated some individual pos EC, abundant deg pos cells in lumen	NT: 38304724
Water treated #8 (Cage 3)	**Lung**: NHA<br>**vAg**: neg	Lung: <LOD; positive
	**Nasal mucosa (HE)**: mild focal NL-dominated rhinitis<br>**vAg**: disseminated patches of pos EC, some individual pos EC	NT: 82700168
Water treated #9 (Cage 3)	**Lung**: NHA, apart from patch of mild pb leukocyte infiltration<br>**vAg**: bronchus and a few bronchioles with large patches to all pos EC	Lung: 48235106; positive
	**Nasal mucosa (HE)**: focal deg and loss of EC, mild NL-dominated rhinitis	NT: <LOD

	**vAg**: mf large patches of pos EC, disseminated some individual pos EC some deg pos cells in lumen	
**Infected #4** (Cage 4)	**Lung**: mild mf pb leukocyte infiltration; mod pb and mild bronchial LC-dominated leukocyte infiltration and scattered deg BEC, many leukocytes incl vacuolated macrophages and deg cells in lumen; mild mf pv leukocyte infiltration; mild mf vasculitis<br>**vAg**: mf patches of alveoli with pos AEC, also with pos deg cells and macrophages; bronchus and several bronchioles with individual and partly large patches of pos BEC and many pos cells in lumen	Lung: 5152977; positive
	**Nasal mucosa (HE)**: epithelium partly flattened, with deg EC; mod NL-dominated rhinitis<br>**vAg**: mf large patches of pos EC, disseminated some individual pos EC	NT: <LOD
Water treated #10 (Cage 4)	**Lung**: mod pb and very mild bronchial leukocyte infiltration, adjacent focal area with activated type II pc, some type II pc/BEC hyperplasia, leukocyte infiltration; mod pv LC-dominated leukocyte infiltration<br>**vAg**: mf, mainly pb patches of alveoli with pos AEC, also with pos deg cells and macrophages; bronchus and several bronchioles with partly large patches of pos BEC	Lung: <LOD; positive
	**Nasal mucosa (HE)**: mild focal NL-dominated rhinitis<br>**vAg**: mf large patches of pos EC, disseminated some individual pos EC	NT: <LOD
Water treated #11 (Cage 4)	**Lung**: NHA<br>**vAg**: several patches of pos EC in bronchus	Lung: 22432; positive
	**Nasal mucosa (HE)**: mild focal NL-dominated rhinitis<br>**vAg**: mf large patches of pos EC, disseminated some individual pos EC	NT: 204386093
Water treated #12 (Cage 4)	**Lung**: NHAIR, apart from a few bronchioles with occ deg cells<br>**vAg**: rare small patches of alveoli with pos AEC; a few bronchioles with a few patches or almost all BEC pos	Lung: 1411636; positive
	**Nasal mucosa (HE)**: mild NL-dominated rhinitis<br>**vAg**: disseminated mf large patches of pos EC, also in larynx	NT: 89840588
**Infected #5** (Cage 5)	**Lung**: mild mf pb leukocyte infiltration with mod vasculitis of adjacent vessel; mod pb and mild bronchial LC-dominated leukocyte infiltration and scattered deg BEC, abundant leukocytes incl vacuolated macrophages and deg cells in lumen; a few areas with desquamation of AEC and AM and leukocyte infiltration<br>**vAg**: mf patches of alveoli with pos AEC, also with pos deg cells and macrophages, particularly abundant pos cells in areas with desquamative component; bronchus and bronchioles with individual or patches of pos BEC and abundant pos cells in lumen	Lung: 7317851; positive
	**Nasal mucosa (HE)**: focal flattened BEC with occasional deg EC, mild to mod NL-dominated rhinitis<br>**vAg**: neg	NT: 1035961
Water treated #13	**Lung**: bronchioles with occ deg EC and deg cells in lumen	Lung: 1163271; positive

(Cage 5)	**vAg**: several patches of alveoli with pos AEC, also with pos deg cells and macrophages; many bronchioles with individual or patches to all pos BEC and some pos cells in lumen	
	**Nasal mucosa (HE)**: mod focal NL-dominated rhinitis<br>**vAg**: disseminated large patches of pos EC	NT: 133751500
Water treated #14 (Cage 5)	**Lung**: NHA<br>**vAg**: neg, apart from patches and individual pos intact EC in bronchus	Lung: <LOD; positive
	**Nasal mucosa (HE)**: individual deg EC<br>**vAg**: large patches of pos EC (mainly apical mucosa)	NT: 14874290
Water treated #15 (Cage 5)	**Lung**: a few bronchioles with occ deg EC in lumen<br>**vAg**: one large and a few smaller patches of alveoli with pos AEC, also with pos deg cells and macrophages; several bronchioles with large patches or all pos BEC	Lung: 21679342; positive
	**Nasal mucosa (HE)**: mild NL-dominated rhinitis<br>**vAg**: neg	NT: 104759676
**Infected #6** (Cage 6)	**Lung**: mf to coalescing, mainly pb leukocyte infiltrates with activated type II pc; some focal areas with desquamation of AEC and AM, some deg cells and vacuolated macrophages, mild type II pc/BEC hyperplasia; mild to mod vasculitis and pv LC-dominated leukocyte infiltration, mild periarterial oedema; mild pb and bronchiolar LC-dominated leukocyte infiltration and scattered deg BEC and mild BEC hyperplasia; mf mod acute alveolar oedema, focal pleural mesothelial cell activation<br>**vAg**: mf, partly coalescing patches of alveoli with pos AEC, also with pos deg cells and macrophages; individual and small patches of pos intact BEC	Lung: 289266; positive
	**Nasal mucosa (HE)**: deg EC and flattened epithelium, mod NL-dominated rhinitis<br>**vAg**: disseminated individual and small patches of pos EC; abundant pos deg cells in lumen	NT: 183470
Nafamostat treated #1 (Cage 6)	**Lung**: focal areas with desquamation of AEC and AM, leukocyte infiltration and activated type II pc<br>**vAg**: neg, apart from two weakly pos macrophages in one focal area	Lung: <LOD; positive
	**Nasal mucosa (HE)**: NHA<br>**vAg**: neg	NT: <LOD
Nafamostat treated #2 (Cage 6)	**Lung**: a few, partly pb focal areas with leukocyte infiltrates, activated type II pc and minimal type II pc/BEC hyperplasia<br>**vAg**: neg	Lung: <LOD; positive
	**Nasal mucosa (HE)**: NHA<br>**vAg**: neg	NT: 28849
Nafamostat treated #3 (Cage 6)	**Lung**: a few, partly pb focal areas with leukocyte infiltrates, activated type II pc and minimal type II pc/BEC hyperplasia<br>**vAg**: neg	Lung: <LOD; positive
	**Nasal mucosa (HE)**: NHA	NT: <LOD

	**vAg**: neg	
**Infected** #7<br>(Cage 7)	**Lung**: mf to coalescing, mainly pb leukocyte infiltrates with activated type II pc, in some focal areas desquamation of AEC and AM, some deg cells and vacuolated macrophages; mild vasculitis; mild pb and bronchiolar LC-dominated leukocyte infiltration and scattered deg BEC<br>**vAg**: multiple patches of alveoli with pos AEC, also with pos deg cells and macrophages; individual and small patches of intact BEC	Lung: 459247;<br>positive
	**Nasal mucosa (HE)**: deg EC and flattened nasal epithelium, mod NL-dominated rhinitis<br>**vAg**: disseminated individual and small patches of pos EC; abundant pos deg cells in lumen	NT: 79173
Nafamostat treated #4<br>(Cage 7)	**Lung**: mf to coalescing large areas with leukocyte infiltrates, activated type II pc and type II pc/BEC hyperplasia<br>**vAg**: neg	Lung: <LOD;<br>negative
	**Nasal mucosa (HE)**: NHA<br>**vAg**: neg	NT: <LOD
Nafamostat treated #5<br>(Cage 7)	**Lung**: one focal pb area with leukocyte infiltrates, activated type II pc and type II pneumocyte/BE hyperplasia<br>**vAg**: neg	Lung: <LOD;<br>positive
	**Nasal mucosa (HE)**: NHA<br>**vAg**: neg	NT: <LOD
Nafamostat treated #6<br>(Cage 7)	**Lung**: NHA<br>**vAg**: neg	Lung: <LOD;<br>negative
	**Nasal mucosa (HE)**: mild focal NL-dominated rhinitis<br>**vAg**: neg	NT: <LOD
**Infected** #8<br>(Cage 8)	**Lung**: mf pb leukocyte infiltrates with activated type II pc and mild vasculitis of adjacent vessel; mod pb and mild bronchial LC-dominated leukocyte infiltration and scattered deg BEC, abundant leukocytes incl vacuolated macrophages and deg cells in lumen<br>**vAg**: some patches of alveoli with pos AEC, also with pos deg cells and macrophages; bronchus with several pos BEC, many bronchioles with multiple patches up to all pos BEC and abundant pos cells in lumen	Lung: 455274;<br>positive
	**Nasal mucosa (HE)**: deg EC and flattened nasal epithelium; severe NL-dominated rhinitis<br>**vAg**: induvial and small patches of pos EC; abundant pos deg EC and pos cells in lumen	NT: 947579
Nafamostat treated #7<br>(Cage 8)	**Lung**: some pb foci with type II pc/BEC hyperplasia; several focal areas with leukocyte infiltration, also foci with aggregates of vacuolated macrophages in alveolar lumina; very mild focal pv leukocyte infiltration<br>**vAg**: neg	Lung: <LOD;<br>positive
	**Nasal mucosa (HE)**: NHA<br>**vAg**: neg	NT: <LOD

Nafamostat treated #8 (Cage 8)	**Lung**: several pb foci with type II pc/BEC hyperplasia and leukocyte infiltration, also foci with aggregates of vacuolated macrophages in alveolar lumina; very mild focal pv leukocyte infiltration<br>**vAg**: neg, apart from two weakly pos cells (macrophages/type II cells) in one pb focus	Lung: <LOD; negative
	**Nasal mucosa (HE)**: mod focal NL-dominated rhinitis<br>**vAg**: neg	NT: <LOD
Nafamostat treated #9 (Cage 8)	**Lung**: several pb foci with activated type II pc, large syncytial cells and leukocyte infiltration, some with type II pc/BEC hyperplasia, some with aggregates of vacuolated macrophages in alveolar lumina; mild to mod mf pv leukocyte infiltration<br>**vAg**: neg	Lung: <LOD; negative
	**Nasal mucosa (HE)**: NHA<br>**vAg**: neg	NT: <LOD
**Infected #9** (Cage 9)	**Lung**: mf mild to mod pb and mild bronchiolar LC-dominated leukocyte infiltration, scattered deg BEC and vasculitis; a few, mainly pb leukocyte infiltrates with activated type II pc; bronchiole with leukocytes incl vacuolated macrophages and deg cells in lumen; mild mf pv leukocyte infiltration<br>**vAg**: several patches of alveoli with pos AEC, also with pos deg cells and macrophages; bronchus with several pos EC, several bronchioles with multiple patches or all pos BEC and many pos cells in lumen	Lung: 7994668; positive
	**Nasal mucosa (HE)**: mod focal NL-dominated rhinitis<br>**vAg**: disseminated patches of pos EC, some individual pos EC	NT: 73405
Nafamostat treated #10 (Cage 9)	**Lung**: NHA<br>**vAg**: neg	Lung: <LOD; negative
	**Nasal mucosa (HE)**: mod focal NL-dominated rhinitis<br>**vAg**: neg	NT: <LOD
Nafamostat treated #11 (Cage 9)	**Lung**: NHA<br>**vAg**: neg	Lung: <LOD; negative
	**Nasal mucosa (HE)**: NHA<br>**vAg**: neg	NT: 1023042
Nafamostat treated #12 (Cage 9)	**Lung**: NHA<br>**vAg**: neg	Lung: <LOD; negative
	**Nasal mucosa (HE)**: NHA<br>**vAg**: neg	NT: 25332
**Infected #10** (Cage 10)	**Lung**: mf mild to mod pb and mild bronchiolar LC-dominated leukocyte infiltration and scattered deg BEC, bronchus and some bronchioles with abundant leukocytes incl vacuolated macrophages and deg cells in lumen; mf mild to mod vasculitis<br>**vAg**: mf, partly coalescing patches of alveoli with pos AEC, also with pos deg cells and macrophages; bronchus and most bronchioles with multiple patches or all pos BEC and abundant pos cells in lumen	Lung: 20027552; positive

	**Nasal mucosa (HE)**: deg EC and flattened nasal epithelium; marked NL-dominated rhinitis<br>**vAg**: induvial and patches of pos EC; abundant pos deg EC and cells in lumen	NT: 135559
Nafamostat treated #13 (Cage 10)	**Lung**: mf to coalescing, mainly pb leukocyte infiltrates with activated type II pc; mild pb and very mild bronchiolar LC-dominated leukocyte infiltration and scattered deg BEC, mild BEC hyperplasia; mild mf pv lymphocyte infiltration<br>**vAg**: neg	Lung: <LOD; negative
	**Nasal mucosa (HE)**: NHA<br>**vAg**: neg	NT: <LOD
Nafamostat treated #14 (Cage 10)	**Lung**: mf to coalescing, mainly pb leukocyte infiltrates with activated type II pc, often also with type II pc/BEC hyperplasia; mild pb and very mild bronchiolar LC-dominated leukocyte infiltration, scattered deg BEC and mild BEC hyperplasia, one bronchiole with leukocytes in lumen<br>**vAg**: neg	Lung: <LOD; negative
	**Nasal mucosa (HE)**: NHA<br>**vAg**: neg	NT: <LOD
Nafamostat treated #15 (Cage 10)	**Lung**: mf, mainly pb leukocyte infiltrates with activated type II pc, focal desquamation of AEC and AM; mod mf acute alveolar oedema; very mild bronchiolar LC-dominated leukocyte infiltration and scattered deg BEC; mod mf pv LC-dominated leukocyte infiltration; mild vasculitis<br>**vAg**: neg	Lung: <LOD; negative
	**Nasal mucosa (HE)**: NHA<br>**vAg**: neg	NT: <LOD

Legend: AEC – alveolar epithelial cells; AM – alveolar macrophages; AT – airborne transmission; BEC – bronchiolar epithelial cells; deg – degenerate; d – day; EC – epithelial cells; HE – histological features assessed in a hematoxylin-eosin stained section; in – intranasal; LC – lymphocyte; mf – multifocal; mode – moderate; neg – negative; NHA – no histological abnormality; NL – neutrophils; NT – nasal turbinates; LC – lymphocytes; pb – peribronchiolar; pc – pneumocytes; pos – positive; pv – perivascular; vAg – viral antigen

[1] PCR: Copies of viral N-RNA/μg of RNA relative to 18S; plaque assay: SARS-CoV-2 viral titre (PFU/mL)

[2] leukocyte infiltration/leukocyte infiltrates: if not further specified, this implies macrophages, fewer lymphocytes and variable numbers of neutrophils
